# It’s Dead! Can Postbiotics Really Help Performance and Recovery? A Systematic Review

**DOI:** 10.3390/nu16050720

**Published:** 2024-03-01

**Authors:** Chad M. Kerksick, Jessica M. Moon, Ralf Jäger

**Affiliations:** 1 Exercise and Performance Nutrition Laboratory, Department of Kinesiology, College of Science, Technology, and Health, Lindenwood University, St. Charles, MO 63301, USA; 2 Exercise Physiology, Intervention, and Collaboration Lab, School of Kinesiology and Rehabilitation Sciences, University of Central Florida, 12494 University Blvd, Orlando, FL 32816, USA; jessica.moon@ucf.edu; 3 Increnovo, LLC, Whitefish Bay, WI 53217, USA; ralf.jaeger@increnovo.com

**Keywords:** biotic, exercise, athletes, sport nutrition, performance, recovery

## Abstract

In recent years, postbiotics have increased in popularity, but the potential relevancy of postbiotics for augmenting exercise performance, recovery, and health is underexplored. A systematic literature search of Google Scholar and PubMed databases was performed with the main objective being to identify and summarize the current body of scientific literature on postbiotic supplementation and outcomes related to exercise performance and recovery. Inclusion criteria for this systematic review consisted of peer-reviewed, randomized, double-blind, and placebo-controlled trials, with a population including healthy men or women >18 years of age. Studies required the incorporation of a postbiotic supplementation regimen and an outcome linked to exercise. Search terms included paraprobiotics, Tyndallized probiotics, ghost biotics, heat-killed probiotics, inactivated probiotics, nonviable probiotics, exercise, exercise performance, and recovery. Only investigations written in English were considered. Nine peer-reviewed manuscripts and two published abstracts from conference proceedings were included and reviewed. Supplementation periods ranged from 13 days to 12 weeks. A total of 477 subjects participated in the studies (*n* = 16–105/study) with reported results spanning a variety of exercise outcomes including exercise performance, recovery of lost strength, body composition, perceptual fatigue and soreness, daily logs of physical conditions, changes in mood states, and biomarkers associated with muscle damage, inflammation, immune modulation, and oxidative stress. Early evidence has provided some indication that postbiotic supplementation may help to support mood, reduce fatigue, and increase the readiness of athletes across several weeks of exercise training. However, more research is needed to further understand how postbiotics may augment health, resiliency, performance, and recovery. Future investigations should include longer supplementation periods spanning a wider variety of competitive athletes and exercising populations.

## 1. Introduction

Probiotics are live microorganisms that, when administered in adequate amounts, confer a health benefit on the host [1]. A long and growing list of potential benefits exists for probiotic use including improvements in gut health [2], mood [3,4], stress, depression [4] and other aspects of mental health [4,5], sleep quantity and quality [3,6], immune system function [7], oral health [8,9], management of allergies [10], and cardiovascular health [11]. In brief, probiotics have the ability to improve health, as they can favorably regulate the immune system by reducing intestinal pH, exclude pathogens, enhance the integrity of the intestinal barrier, and enhance gut microbial diversity, which subsequently contributes to further health benefits by reducing inflammation modulating the immune system [2,12,13,14].

In competitive and noncompetitive exercising populations, evidence suggests that regular exercise also serves as a modulator of gut microbiota composition and function [15,16,17,18]. Moreover, evidence also suggests that differences in dietary composition and fitness status of exercising individuals may also invoke differences in the gut microbiome composition and function [16,19]. Recently, a position stand on probiotics by the International Society of Sports Nutrition highlighted several areas where probiotics may work to aid the training and competition demands of athletes [20]. The highlighted areas from this review include the strengthening or modulation of the immune system to avoid or minimize the impact of illness and infections [21], potential improvements in recovery [22,23], and the improvement or enhancement in the maintenance of gut permeability [24,25]. In addition to these areas, there is widespread interest regarding the potential of probiotics to positively influence exercise performance [26]. In this respect, previous studies have demonstrated that supplementation with specific probiotic strains can increase running time to exhaustion in the heat [27] and impact strength, endurance, and body composition [28,29,30]. In addition to overt ergogenic outcomes, probiotics may also favorably impact an athlete’s overall training and performance through heightened mood and improved recovery [20,23]. Thus far, evidence has rapidly accumulated to support the notion that probiotic use may positively impact several aspects of human health and favorably influence a variety of aspects related to exercise training and performance. While more research is needed to further define the specific species, strains, and dosing regimens that most reliably interact with factors related to an athlete’s training and performance [31], current evidence strongly supports the idea that probiotics are an important consideration for athletes looking to support their health and training.

For years, cell viability was considered to be one of the most important, if not the most important, characteristic of a probiotic responsible for conferring health benefits. However, research spanning several years has indicated the potential for nonviable cells, microbes, metabolites, and cellular components to exhibit functional properties that may impact health [32]. Supporting this notion, the International Scientific Association of Probiotics and Prebiotics (ISAPP) recently proposed what is likely the most referenced definition of a postbiotic—a “preparation of inanimate microorganisms and/or their components that confers a health benefit” [33]. Notably, due to the long-standing belief that cell viability is critical, debate has ensued regarding a proper definition, with a full discussion of this issue being beyond the scope of this review [34,35,36].

Ultimately, the term postbiotic is derived from the Greek for ‘post’, which means after, and ‘biotic’, which means life. When considered further, this term expands on the established lineage of prebiotic, probiotic, and symbiotic, which converge with reference to microbes or the microbial components they produce. In brief, the term postbiotic refers to substances of microorganism origin that are no longer alive, making them inanimate, dead, or inactivated. Nevertheless, postbiotics extend well beyond the simple definition of a ‘dead probiotic’ and can be inanimate whole or parts of cells and can include large or small fragments of the original microbe. For example, heat-killed probiotics can contain inactive bacterial cells and/or metabolites that are produced by live probiotics such as exopolysaccharides, peptidoglycan, lipoteichoic acid, short-chain fatty acids, or amino acids [37], which have been shown to effectively modulate the gut microbiome and improve aspects of health [38,39,40].

Regardless of whether a universal definition can be established, no one can question that conferred health benefits are associated as a hallmark component of the probiotic definition and that these benefits are not dependent upon the viability of the cells [32,41,42]. Likewise, a review by Adams highlighted that, in some instances, dead or inactivated cells can cause robust responses that are on a similar scale and scope as live versions of the same cells [41]. In this respect, a previous study demonstrated that nonviable bacteria and associated fractions could better traverse through or around mucus and stimulate several cells more consistently than live cells of the same type [43] while also exhibiting similar potential to positively impact health when compared to live cells [44,45].

Moreover, similar to probiotics, the outcomes and the extent of their impact may vary depending on the specific strain of postbiotic consumed. In this regard, Pique and colleagues [46] reviewed the observed health benefits of postbiotic administration and found that heat-killed (Tyndallized) probiotics could exert favorable outcomes in gastrointestinal, dermatological, and respiratory conditions and disease. Additionally, the modulation of other health benefits such as cold and acute illnesses [47], gut function [48], immunomodulatory considerations [49], and bolstered outcomes surrounding stress and sleep quality [50] and vitality of pre-term infants [51] were also reported. Methods to inactivate bacterial cells aim at maintaining their surface structure and include heat, sonification, chemical treatment, and UV irradiation. The method as well as the processing conditions that influence the activity of the resulting postbiotic are currently not sufficiently described in most publications [52].

As research continues to deepen our understanding of the mechanisms of action for postbiotics, several practical reasons have emerged as to why utilizing postbiotic formulations of probiotics strains may be useful [14]. First, probiotic survival during shelf life, manufacturing, and digestion is limited due to sensitivity to heat or water, and for these reasons, most probiotic cells do not survive the passage through the gastrointestinal tract due to low pH in the stomach or bile acids. Second, the survivability of probiotics in liquids such as sports beverages is very limited, eliminating several delivery formats of ‘biotics’ popular with athletes. You cannot kill what is already dead, and concern persists that the dead cells may evoke an unfavorable physiological response upon ingestion or negatively impact organoleptic considerations related to human ingestion. Next, a third distinct advantage of postbiotics is the ease through which postbiotics can be produced and their ability to be standardized for commercial applications. Finally, and probably the most significant advantage, postbiotics have a longer shelf life and are less susceptible to degradation from changes in ambient conditions, which makes them much easier to store and transport. In addition, postbiotics are favored for consumption as they offer improved safety in terms of food quality and are less risky for immune-compromised individuals [53,54]. Further, postbiotics do not translocate from the gut lumen to the blood, which eliminates the potential for them to acquire and transfer antimicrobial resistance genes [42,46,51]. Considering these factors, the long-term viability of the live bacterial cells found in probiotics will inevitably become damaged or killed [53], which reduces the total number of available and viable bacteria, subsequently increasing the proportion of cells that are nonviable. Pragmatically speaking, one must consider at what point does the loss of viability become a concern in terms of how many viable cells are being delivered and at what point the proportion of nonviable cells may be responsible for the observed outcome rather than the viable cells. In short, for acute probiotic studies, this may be less concerning; however, in the case of longer clinical trials, the actual dosage of live bacteria cells delivered may change drastically from start to finish. Despite this, utilizing postbiotics may effectively overcome many of these challenges.

Considering the well-established and ever-maturing scientific literature base examining probiotics and exercise and the rapidly growing appreciation and inquiries related to postbiotics, the need to summarize the current literature base exploring the impact of postbiotic administration in exercising individuals is warranted. Therefore, the primary aim of this systematic review is to summarize and evaluate the current available literature on the potential impact of postbiotics in sport.

## 2. Materials and Methods

### 2.1. Study Selection

**Figure 1:**
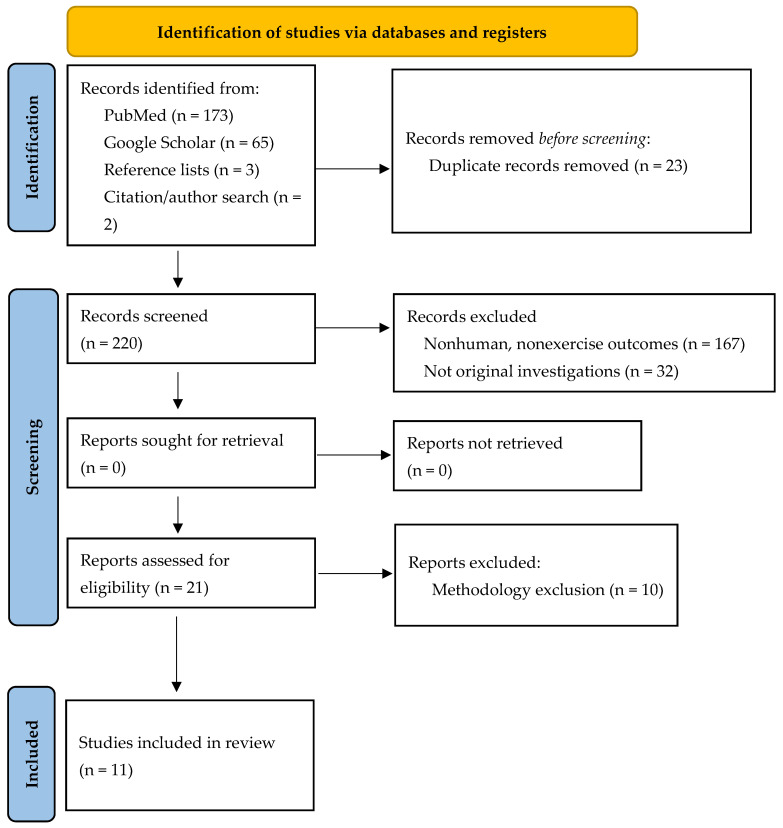
PRISMA diagram.

A computerized systematic literature search of the electronic databases PubMed and Google Scholar, from formation up to 19 January 2024, was conducted to identify studies that examined the impact of postbiotic supplementation, with or without an exercise intervention, on outcomes specific to exercise performance, recovery, and biomarkers associated with muscle immune function, inflammation, and oxidative stress. Relevant terms were included in the search strategy to identify potentially pertinent studies as follows: (paraprobiotics OR Tyndallized probiotics OR ghostbiotics OR heat-killed probiotic OR inactivated probiotic OR nonviable probiotics) and (exercise OR exercise performance OR recovery OR exercise recovery) with no restrictions. In addition, the studies to be included were assessed to identify whether there was appropriate relevance for the purpose of the current systematic review, while the reference lists of all published works deemed appropriate were examined to potentially identify additional eligible studies. No searching was completed by hand. Further, the search, study selection, and reporting procedures were independently performed by two authors (CK and JM). Figure 1 presents a flow diagram, using the Preferred Reporting Items for Systematic Reviews and Meta-Analyses (PRISMA), of the search process [55]. This systematic review has not been publicly registered.

### 2.2. Inclusion and Exclusion Criteria

The following inclusion criteria were considered when evaluating the potentially relevant investigations: (1) Types of Investigations: peer-reviewed, randomized, double-blind, placebo-controlled trials; (2) Types of Participants: healthy, men or women, aged 18 and older; (3) Type of Supplemental Intervention: consumption of any of the following: 1. paraprobiotics, 2. Tyndallized probiotics, 3. ghostbiotics, 4. heat-killed probiotics, 5. inactivated probiotic, 6. nonviable probiotics; (4) Types of Outcomes: at least one outcome of exercise, exercise performance, or exercise recovery were reported in the articles; (5) Language: articles written in English; (6) Types of Design: pre/post-measurements surrounding postbiotic intervention and a control group. In contrast, exclusion criteria for the investigations included the following: (1) articles or abstracts of which no full text was found; (2) nonhuman studies; (3) case reports, reviews, and meta-analyses; and (4) studies conducted on children, adolescents, pregnant women, or patients with chronic illnesses or diseases. Studies using just metabolites and not containing inanimate cells and/or cell fragments were excluded.

### 2.3. Data Extraction

To avoid bias, a standardized electronic document was used to extract the data from all investigations that were deemed eligible after screening. The data collected were as follows: (1) name of the first author and publication year, (2) sample size, (3) age and sex of participants included, (4) study design, (5) postbiotic species/strain, dosing, and duration, (6) exercise intervention (if applicable), (7) exercise performance or recovery endpoint outcomes assessed, and (8) a summary of the investigation’s findings. All results were summarized and are presented in Table 1.

## 3. Results

Using the criteria outlined in Figure 1, a total of 243 articles were identified: (PubMed, *n* = 173; Google Scholar, *n* = 65; reference lists, *n* = 3; citation/author search, *n* = 2). Duplicate records (*n* = 23) were removed; therefore, 220 articles were screened. Of these, 167 were excluded because they were either conducted in a nonhuman model or the outcomes were not aligned to exercise, performance, or recovery. An additional 32 publications were excluded because they were not original investigations. This resulted in 21 papers being assessed for eligibility, where an additional 10 papers were excluded due to some aspect of the methodology being violated. A total of 11 papers were reviewed and included as part of this systematic review. Due to the small number of articles retrieved, a narrative approach to summarizing the current outcomes was completed and all results have been organized into a summary table (Table 1). No risk of bias, assessments of certainty, or sensitivity analysis were completed.

## 4. Discussion

### 4.1. Exercise + Postbiotics: Exercise Performance + Recovery

In many aspects of sports nutrition, the potential for ergogenic modulation is one of the most popular and sought-after outcomes for any exercise, diet, or supplemental intervention. As one reviews the initial studies examining postbiotic supplementation and sport, many of the available investigations have examined the potential of postbiotics to enhance performance. In this respect, a small number of studies have begun to explore the ergogenic potential of postbiotics, with more anticipated to be published in the coming years. As further research is completed, our understanding of postbiotics’ ergogenic potential, as well as how these outcomes may differ from other supplemental or ergogenic approaches, will continue to evolve.

One investigation by Hoffman and colleagues [56] investigated the potential for an inactivated probiotic strain (*Weizmannia coagulans* GBI-30 6086) to impact human performance and recovery. During the trial, 16 soldiers stationed on base, experiencing the same training regimen, were supplemented in a randomized, double-blind format with either a placebo or 1.0 × 10^9^ inactivated *W. coagulans* GBI-30 6086 cells per day for 14 days. Before and after the supplementation protocol, changes in performance (vertical jump, muscle endurance, shuttle runs, and casualty drag), hormone concentrations (testosterone and cortisol), creatine kinase, and inflammatory cytokines were evaluated. While no statistically significant changes were observed for any measured outcomes, statistical trends (*p* = 0.050–0.100) and a magnitude-based inference approach suggested that supplementation likely enhanced lower body power and a casualty drag (a military-specific measure of performance) and likely enhanced the anti-inflammatory cytokine interleukin-10. No other changes in endocrine, cytokine, or muscle damage markers were observed. Furthermore, due to the inability of some of the soldiers to complete the prescribed post-testing assessments and the already small sample size, the statistical power to determine statistically significant changes was likely reduced, which is supported by the large magnitude of changes observed in conjunction with the statistical trends for some variables being impacted by supplementation.

Using the same inactivated bacterial strain, Hagele et al. [59], in abstract form, presented the results of an investigation that randomized 76 healthy, resistance-trained males to either a placebo, 1 × 10^9^ CFU/day live cells of *W. coagulans* GBI-30 6086, or 1 × 10^9^ cells/day of heat-killed *W. coagulans* GBI-30 6086 for 14 days prior to completing a stressful bout of lower-body exercise. Before and after the exercise bout, participants were evaluated for changes in strength and power production using isometric mid-thigh pulls, counter-movement jumps, and isokinetic dynamometry. When compared to PLA, neither the live cells nor heat-killed cells of this strain were found to modulate performance under this investigative model.

In 2022, Lee et al. [57] published what may be the first investigation to directly compare, in a head-to-head fashion, a probiotic (live cells) and postbiotic (heat-killed) version of the same bacterial strain. In this investigation, 105 healthy adults were randomized to consume, in a double-blind fashion, either a placebo, 2 × 10^10^ CFU/day of live cells of *Lacticaseibacillus paracasei* PS23, or heat-killed *L. paracasei* PS23 at a dosage of 1 × 10^10^ cells/day. After six weeks of supplementation, each participant completed 100 maximal vertical jumps to induce muscle damage. Exercise performance and biomarkers indicative of muscle damage, oxidative stress, and inflammation were evaluated prior to and 3, 24, and 48 h after completion of the damaging exercise protocol. Furthermore, to evaluate functional performance, participants completed vertical jumps on force plates, isometric mid-thigh pulls, and Wingate anaerobic capacity tests. The results indicated that both live and heat-killed cells of *L. paracasei* PS23 were responsible for slowing the rate at which force development was lost throughout a vertical jump following the damage bout while also facilitating faster recovery when compared to the placebo group. Additionally, both live and heat-killed cells were able to avert the loss of peak force production 3 and 24 h after the muscle damage session when compared to placebo; however, only the heat-killed cells significantly improved strength recovery after 48 h (when compared to placebo). Similarly, vertical jump height in all groups was reduced following the completion of the muscle damage protocol. When compared to placebo, vertical jump height returned to baseline to a significantly greater extent 24 h after the muscle damage bout when supplementing with both the live and heat-killed *L. paracasei* PS23 cells, while at 48 h post-exercise, only the heat-killed version had reported a further improved recovery of baseline vertical jump height. Furthermore, peak force production was also evaluated using an isometric mid-thigh pull as a performance metric. Post-damaging exercise, supplementation with live cells resulted in less force production lost at 24 and 48 h when compared to the placebo, while the group supplementing with heat-killed cells was greater than the placebo at all measured time points. Finally, during the anaerobic capacity assessment, groups supplementing with both the live and heat-killed cells were able to better maintain mean and peak power production potential when compared to placebo. Moreover, only the heat-killed cells supported significantly lower reductions in Wingate fatigue index 24 h after the damage bout when compared to placebo, while both live and heat-killed cells improved fatigue index values 48 h after.

Soon thereafter, another investigation was published comparing, in a head-to-head fashion, the impact of supplementation with live or heat-killed cells of *Lactiplantibacillus plantarum* TWK10 [63]. In this study, 53 healthy adults were randomly divided and supplemented for six weeks with either a placebo, 3 × 10^11^ CFU/day of viable (live) cells of *L. plantarum* TWK10, or heat-killed cells of the same strain (also at a dosage 3 × 10^11^ cells/day). Both groups experienced similar improvements in exercise performance as assessed by a running time to exhaustion test at 85% VO_2_Max. In response to an exercise stimulus, glucose, lactate, and ammonia were lower than placebo, but supplementation with live cells resulted in significantly lower levels when compared to heat-killed cells. Interestingly, the inflammatory response to the exercise bout (as measured by neutrophil-to-lymphocyte and lymphocyte-to-platelet ratio) was increased only after supplementation with the heat-killed cells.

Finally, a study using a heat-killed version of *L. plantarum* TWK10 was published by Cheng et al. [62], which sought to determine the impact of six weeks of supplementation with either a placebo or 3 × 10^10^ heat-killed cells of *L. plantarum* TWK10 in 30 healthy males. No exercise program was provided as part of this study; rather, each participant was supplemented with their assigned supplement and was evaluated for changes in endurance exercise performance, muscle mass, body composition, and muscle stress and fatigue before and following the supplementation period. In the participants that supplemented with the postbiotic, endurance performance, grip strength, and muscle mass were all increased when compared to placebo. In addition to these changes, lactate and ammonia levels (metabolic indicators of stressful exercise) were reduced in the postbiotic group when compared to placebo.

The literature on the use of postbiotics and their impact on exercise performance outcomes is both limited and diverse. As more research is conducted employing diverse training patterns and performance outcomes, and on a wider variety of athletes, our understanding of the ergogenic potential for postbiotics to modulate performance will also be refined. As with other areas of inquiry involving exercise and nutrition interventions, the need for researchers to conduct well-controlled investigations is extremely important. In this regard, the inclusion of consistent, detailed, and standardized reporting of the doses administered throughout supplementation trials is integral. Both of Lee et al.’s studies in 2022 are extremely valuable in terms of providing a well-conducted examination that directly compares live and heat-killed cells of the same strain to placebo and to each other. As such, the heat-killed version of TWK10 has amassed a small number of early findings that suggest it may be a positive modulator of human performance. As more information about postbiotics and their relevancy for sporting populations accumulates and is eventually disseminated, a likely and common question from researchers, athletes, and sports practitioners will inevitably be how heat-killed versions of a bacterial strain may, or may not, afford additional benefits. Subsequently, this will lead to further questions as to why they should, or should not, include them in their daily regimen to support performance outcomes and maximize their recovery potential.

### 4.2. Exercise + Postbiotics: Muscle Damage and Recovery

Another area of research of interest for postbiotics involves the acute administration of dietary interventions to mitigate the responses athletes experience following the completion of acute bouts of damaging, stressful exercise [66,67]. In this respect, many studies that have examined aspects of exercise performance have also assessed aspects of exercise recovery. For example, Lee et al. [57] reported changes in creatine kinase and myoglobin, two established biomarkers of muscle damage, following completion of 100 maximal vertical jumps and supplementation for six weeks with either a placebo, 2 × 10^10^ CFU/day of live cells of *Lacticaseibacillus paracasei* PS23, or heat-killed *L. paracasei* PS23 at a dosage of 1 × 10^10^ cells/day. Supplementation with either formulation resulted in reduced creatine kinase levels 24 and 48 h post-exercise compared to placebo, while changes in myoglobin were lower than placebo at three and 48 h after damage in both live and heat-killed cell groups.

Moreover, in an abstract presented by Holley et al. [60], muscle damage in 76 healthy, resistance-trained males following a stressful, damaging bout of lower-body exercise and supplementation for 14 days with a placebo, 1 × 10^9^ CFU/day live cells, or 1 × 10^9^ cells/day of heat-killed *W. coagulans* GBI-30 6086 was assessed via changes in creatine kinase, myoglobin, soreness, and perceived recovery. Soreness, assessed via pain–pressure threshold on a digital algometer, was significantly improved five hours after the exercise bout when heat-killed cells of this strain were consumed compared to placebo. Furthermore, when compared to the placebo, no differences were identified in perceptions of recovery when heat-killed cells were provided, while consistent changes in perceptions of recovery were identified when live cells were provided in participants. A direct comparison between live cells and heat-killed was not performed. Changes in muscle damage were also evaluated by evaluating circulating changes in creatine kinase and myoglobin. No differences were identified between the placebo and the heat-killed versions of *W. coagulans* GBI-30 6086, while supplementation with live cells reduced creatine kinase levels immediately and 5 h post-exercise; differences between the two conditions tended to be different 1, 2, 24, and 48 h post-exercise.

As previously mentioned, prolonged high-intensity exercise increases the risk of upper respiratory tract infections (URTI), which are common among athletes [68,69]. Previous studies examining heat-killed probiotics have reported the ability of heat-killed versions to bolster immunity defense [70], while a few studies have examined the potential of heat-killed probiotic strains to impact immunity in athletes who were regularly undergoing high-intensity exercise training. In what is likely the first study published that explored any outcome connected to acute exercise with a postbiotic, Sashihara and colleagues [64] supplemented 44 Japanese track and field athletes for four weeks with a placebo, 1 × 10^10^ heat-killed *Lactobacillus gasseri* OLL2809 cells alone, or in combination with 900 mg of alpha-linolenic acid. Before and following the four-week supplementation period, participants had their blood drawn prior to and 30 min after completing a 60 min bout of exercise while additionally completing a mood state questionnaire and a daily log of cold and gastrointestinal symptoms. The results indicated that treatment with the heat-killed probiotic prevented the reduction in natural killer cell activity that is associated with strenuous exercise and worked to improve the mood of participants surrounding the acute exercise bout. Additionally, postbiotic supplementation alleviated minor resting fatigue, which has the potential to help support the mental and physical health of the athletes.

Komano and colleagues [58] randomly supplemented 51 university club athletes (~20 years of age) in a double-blind format with either a cornstarch PLA or 1 × 10^11^ cells of heat-killed *Lactococcus lactis* JCM 5805 for 13 days while they completed their typical high-intensity training sessions for their sport. Antiviral response indicators, subjective indicators of URTI, and markers of muscle damage (creatine kinase and lactate dehydrogenase) and stress (adrenaline and cortisol) were evaluated before and after supplementation. After 13 days of supplementation with *L. lactis* JCM 5805, antiviral response indicators (CD86) increased alongside a reduced number of days where URTI infection symptoms were reported. In addition, a reduction in the cumulative number of days of fatigue was observed with supplementation, while no changes were observed in creatine kinase, lactate dehydrogenase, adrenaline, or cortisol.

Following this, Komano and investigators [65] conducted another investigation in which they divided 37 participants into two groups who took either a placebo or capsules containing 1.0 × 10^11^ cells of *Lactococcus lactis* JCM 5805 for 14 days while continually exercising. Blood samples were collected to evaluate changes in immune activity (CD86 from plasmacytoid dendritic cells, pDCs), and fatigue was assessed before, throughout, and following the supplementation regimen. Accumulation of pDCs increased, alongside significantly fewer days of fatigue in those supplemented with *L. lactis* JCM 5805 when compared to a placebo after 15 days. Acute measures in response to a 2 h exercise after 15 days of supplementation were also assessed. It was found that supplementation with *L. lactis* JCM 5805 lowered autonomic fatigue, leading the authors to conclude that supplementation with the postbiotic *L. lactis* JCM 5805 alleviated the accumulation of fatigue and increased immune component activity. Overall, these results align with what has been observed in nonexercising populations, providing preliminary evidence that postbiotic versions of the *L. lactis* JCM 5805 strain maintain some of its observed efficacy for supporting immune function and that these benefits may be extended to athletes participating in regular exercise training.

Another investigation completed on young, competitive university athletes was published by Sawada and colleagues. In this study [39], the authors supplemented 49 male university runners with either a placebo or 1 × 10^10^ heat-killed cells/day of *L. gasseri* CP2305 for 12 weeks on a daily basis, while they were training for a major university running competition. Physical and mental health were assessed, while blood and fecal samples were collected before and after the supplementation protocol. When compared to placebo, supplementation with the postbiotic facilitated recovery from fatigue and relieved feelings of anxiety and depressive mood. For blood components, hemoglobin concentrations (a key component of oxygen transport) were better maintained in the postbiotic group, while increases in growth hormone were observed. Furthermore, supplementation with the heat-killed cells also increased the alpha and beta diversity that was observed in the fecal microbiome analysis of the stool samples.

The Sawada et al. [39], Komano et al. [58,65], and Sashirara [64] studies all utilized cohorts of competitive athletes and demonstrated the ability of postbiotics to help offset fatigue, illness, infection, and negative changes in mood and depression. Collectively, these results are particularly valuable as the current postbiotic and sports literature has yet to fully examine the ability of postbiotics to support immune system health across an entire training year. When examining the robust human clinical evidence demonstrating probiotics’ efficacy in preventing illness and mitigating excessive training stress in athletes, adjacent to the initial postbiotic research in nonathletic and clinical populations demonstrating the robust ability to support immune health and prevent the onset and minimize illness duration and severity, the need for more research aggressively investigating these outcomes in athletes is highly warranted.

### 4.3. Exercise + Postbiotics: Immunity, Oxidative Stress, and Inflammation

As mentioned in the previous section, there is likely no greater area of interest and importance for postbiotics to become a central part of an athlete’s daily regimen than their ability to prevent illness or minimize the severity and duration of symptoms. Considering that many different strains of bacteria are inactivated by heating (Tyndallized), which leaves the outer bacterial cell wall intact for it to function as a primary interaction point with host immune cells [53], the potential for postbiotics to positively impact athlete immune health is compelling. Emanating from this line of reasoning, many investigations have evaluated and documented the ability of postbiotics to support health and immune function outside of athletics. For example, a 2023 paper by Sato et al. [71] randomized 200 healthy adults to a placebo or 5 × 10^10^ heat-killed *Lacticaseibacillus paracasei* MCC1849 cells for 24 weeks. Cold symptoms were subjectively evaluated by all participants and the number of days with a stuffy nose and cold-like symptoms, as well as the duration of a stuffy nose, sore throat, and other cold-like symptoms, were significantly reduced in the postbiotic group vs. placebo. Furthermore, following years of culminating research, a firm link has been established between heavy volumes of exercise training and the robustness of the immune system [72]. A well-characterized “inverted J hypothesis” has been described in athletes, stating that initial increases in exercise volume effectively strengthen the immune system, but as exercise and training volume increase, the susceptibility to contracting a cold or suffering from acute illness will rise [72]. As research investigating postbiotics and exercise or exercising populations has begun to appear, a small number of these studies have explored the potential for a postbiotic to mitigate responses routinely observed from immune, inflammatory, or oxidative stress.

In early preclinical research by Jensen et al. [52], inactivated *W. coagulans* GBI-30 6086, the strain used by Hoffman [56] and in preliminary research by Hagele et al. [59] and Holley et al. [60], demonstrated similar immune activation and anti-inflammatory benefits compared to live *W. coagulans* GBI-30 6086 cells. Moreover, continued in vitro efforts clearly highlighted the potential for inactivated *W. coagulans* GBI-30 6086 cells to exhibit potent immune-activating properties. Additionally, human work involving inactivated cells of this strain revealed that 28 days of daily supplementation with 5 × 10^8^ cells increased immune cell and cytokine responses. Furthermore, in 2018, Kalman and Hewlings [61] conducted a randomized, double-blind, placebo-controlled trial that examined the safety and efficacy of consuming inactivated *W. coagulans* GBI-30 6086 cells at rest and after an in vitro lipopolysaccharide (LPS) challenge in response to completing a stressful bout of treadmill exercise. Sixteen healthy males between 18 and 30 years of age were supplemented for 28 days with a placebo or 50 mg of inactivated *W. coagulans* GBI-30 6086. Each participant had vital signs, adverse events, and venous blood collected surrounding completion of a 60 min bout of treadmill activity between 60 and 80% of heart rate reserve. Several immune markers (salivary IgA, complete blood counts, cytotoxic T-cells, and natural killer cells), cortisol, and several pro- and anti-inflammatory cytokines were evaluated before and after supplementation. No between-group differences were observed to suggest that the inactivated cells performed differently from a statistical perspective than placebo. When changes within the inactivated condition were considered, improvements in T cell counts and T cell proportions were observed, which may suggest that the postbiotic reduced the size and duration of the immune “open window”, commonly reported after stressful exercise. Additionally, postbiotic supplementation in this model did not impact circulating levels of inflammatory cytokines up to two hours after completion of one hour of moderate-intensity treadmill activity.

Alternatively, Lee et al. [63] reported significant improvements in two markers of inflammatory response (neutrophil-to-lymphocyte and lymphocyte-to-platelet ratio) following supplementation with a heat-killed version of *L. plantarum* TWK10 for six weeks and after completion of an acute exercise bout when compared to placebo or live cells of the same strain. An additional study examining postbiotic supplementation and inflammatory outcomes in an exercising population was completed by Holley et al. [60]. In this trial, the observed changes in circulating levels of C-reactive protein, IL-6, IL-10, TNF-α, MCP-1, and circulating cell counts were assessed to evaluate the ability of supplementation with live cells and heat-killed cells of *W. coagulans* GBI-30 6086 to mitigate the inflammatory response following muscle-damaging exercise. When compared to placebo, no changes were observed in cell counts, C-reactive protein, and MCP-1 for either form of the bacterial strain. IL-6 concentrations were greater in placebo when compared to heat-killed cells 30 min and 2 h post-damaging exercise, while live cells were lower 5 and 72 h post-exercise. TNF-α concentrations were not different from placebo when heat-killed cells of *W. coagulans* GBI-30 6086 were consumed. IL-10 concentrations (IL-10 is an anti-inflammatory cytokine, thus higher levels are deemed to be favorable) indicated that both heat-killed (5 and 24 h) and live cells (immediate, 2, 5, 24, and 72 h) of *W. coagulans* GBI-30 6086 had lower circulating levels when compared to placebo. This seemingly unfavorable response was not expected and did not align with other studies reported for this strain and other strains.

Oxidative stress is another popular area of research for exercising populations. Currently, few studies involving postbiotics, exercise, and oxidative stress have been reported in the literature. As a result, many studies of this nature are needed to aid in furthering our understanding of how postbiotics may impact these outcomes. From the current literature, changes in oxidative stress (TBARS) were evaluated by Lee et al. [57], along with inflammation via C-reactive protein. Supplementation with either the live or heat-killed version of *L. paracasei* PS23 resulted in reduced levels of circulating TBARS (a marker of lipid peroxidation) at 24 and 48 h post-exercise versus placebo; however, the observed reductions with heat-killed cells were significantly lower than both placebo and with live cells of *L. paracasei* PS23. Likewise, changes in C-reactive protein were found to be significantly lower in both the live and heat-killed cells when compared to placebo. Similarly, heat-killed versions of *L. paracasei* PS23 led to significantly reduced changes in C-reactive protein when compared to the changes observed with live cells of *L. paracasei* PS23 at the 48 h assessment point.

In summarizing the available findings connecting postbiotic use to changes in immune function and inflammatory and oxidative changes while exercising, one must closely evaluate and contextualize the potential value that postbiotics may have for competitive athletes. More specifically the stress of training can induce a compromised state, while the oftentimes intense travel schedules that many athletes will follow can also contribute to immunological and oxidative stress. Notably, all forms of travel, particularly air travel, that traverse multiple time zones can negatively impact sleep and recovery while providing opportunities for opportunistic pathogens to enter and take hold inside an athlete’s body. What results is an athlete with an increased susceptibility to contracting and suffering from illnesses such as common colds, flu, and related upper respiratory infections (URTIs). To date, limited research is available to assist in understanding the modulatory potential of postbiotics for an athlete’s immune system. Certainly, with the enhanced flexibility for postbiotics to be carried during travel without the worry of maintaining optimal storage temperatures, short-shelf lives, and other cell viability roadblocks that arise with probiotics, the potential for postbiotics to support the immune health of athletes is an exciting and promising area for future research.

As identified and discussed throughout this systematic review, the literature base for postbiotic use in athletic populations is extremely limited, and only six different postbiotics have been studied: *Weizmannia coagulans* GBI-30 6086 *Lacticaseibacillus paracasei* PS23, *Lactococcus lactis* JCM 5805, *Lactiplantibaccilus plantarum* TWK10, *Lactobacillus gasseri* CP2305, *Lactobacillus gasseri* LG2809, and *Lactococcus lactis* plasma. While mechanistic considerations for postbiotics have been proposed by Ma and colleagues [73], no research to date has systematically examined the extent to which any of these mechanisms, or any others, may help to explain the efficacy surrounding postbiotic use in athletes. Much like the challenges involving probiotic use in sports, early findings have suggested that strain specificity with postbiotics will continue to be a central issue, while study design considerations including dosing amount and duration should be critical factors considered in future research [74].

Finally, the current limitations surrounding this systematic review were the inclusion of published articles that were only written in English. Beyond that, the reader must understand that the literature base for this topic is still quite small, and with the current interest levels in sport and active nutrition, probiotics, and postbiotics, the number of commercially available postbiotics targeted for sport and scientific investigations involving them will likely increase substantially in the next five years. In this respect and while all results were described according to key content areas of interest in sports and active nutrition, the number of published studies was small. In some instances, only one or two published studies were available for discussion. Thus, more research is needed in nearly every area of postbiotics and sport to help uncover any potential efficacy for postbiotics in sports nutrition applications.

## 5. Conclusions

As further research is completed, our understanding of the health and ergogenic potential of postbiotics to athletes or active individuals, as well as how these outcomes may differ from other supplemental or ergogenic approaches, will continue to evolve. Of the current literature that is available, a wide divergence of study designs, questions, and outcomes is present, resulting in only a small number of studies that have examined commonly applied exercise performance outcomes. In all areas, more research is needed to fully understand the situations in which postbiotics may be used to be most efficacious for exercising individuals looking to improve their health, performance, and recovery. To this point, only three studies to date have directly compared pro- and postbiotic versions of the same strain, *Lactiplantibaccilus plantarum* TWK10, *Lacticaseibacillus paracasei* PS23, and *Weizmannia coagulans* GBI-30 6086, indicating comparable but different activity of the postbiotic compared to the probiotic, and studies combing post- and probiotic to investigate potential additive or even synergistic effects are currently lacking. Dose–response and studies investigating the effect and potential difference in efficacy based on production methods and process conditions are lacking as well. It also stands to reason that as probiotics grow in popularity and are used in confidence by athletes, coaches, and practitioners to support the health, training, and competitive desires of the athlete, so too will the popularity of postbiotics. As previously mentioned, one of the most clearly documented benefits of postbiotics versus many probiotic strains involves their greater shelf life, stability, and resilience to breakdown due to uncontrolled storage conditions. When one layers these advantages over the strenuous and exacting traveling lifestyle of a competitive athlete, the advantages for postbiotic use in athletic populations seem realistic and may in fact be a powerful, pragmatic motivation for postbiotics to become more popular in athletes than their probiotic counterparts. However, more research is needed in nearly all areas before firm conclusions can be made about how and where (and if) postbiotics should become a key part of an athlete’s regimen. A key focus moving forward should be to identify if other advantages exist, particularly those advantages that might relate directly to better health, performance, or recovery.

## Figures and Tables

**Table 1:** Summary table of research studies involving postbiotic supplementation and exercise.

Reference	Participants (*n*, Age, Sex)	Study Design	Supplementation (Duration)	Exercise Intervention	Endpoints	Findings
Hoffman, 2019 [56]	16 male soldiers	RAN DB PLA PAR	1.0 × 10^9^ CFU of inactivated *Weizmannia coagulans* GBI-30 6086 or PLA (14 days)	Same daily protocol as part of soldiers’ self-defense training course	- Physical performance - Inflammatory cytokines - Endocrine response (testosterone, cortisol) - Muscle damage (creatine kinase)	- No change in performance or blood variables - Trends for improved vertical jump power and casualty drag - Trends for improvements in IL-10 and IFNγ
Lee, 2022 [57]	105 healthy males and females between (avg. age of 21.6 years)	RAN DB PLA PAR	PLA vs. 2 × 10^10^ CFU/day live *Lacticaseibacillus paracasei* PS23 (DSM 32322) cells vs. heat-killed *L. paracasei* PS23 (DSM 32322). Equivalent to 1 × 10^10^ cells/day (6 weeks)	100 maximal vertical jumps	- 0, 3, 24, and 48 h after supplementation - Performance (countermovement jumps, isometric mid-thigh pull, Wingate anaerobic capacity) - Muscle damage (creatine kinase and myoglobin) - Inflammatory markers	- Both live and heat-killed *L. paracasei* PS23 slowed muscle strength loss, ↑ anaerobic cycling performance, and ↓ muscle damage markers and inflammation - Heat-killed *L. paracasei* PS23 demonstrated more favorable patterns of change for fatigue, oxidative stress, CRP, and testosterone
Komano, 2018 [58]	51 male sports club athletes	RAN DB PLA PAR	PLA vs. 1 × 10^11^ heat-killed *Lactococcus lactis* JCM 5805 (13 days)	High-intensity exercise as part of sport training program		- CD86 increased in LC-plasma at day 14 - URTI days were lower in LC-plasma vs. PLA - Days of fatigue were lower in LC-plasma vs. PLA - Muscle damage and stress ND between groups
Hagele, 2023 (Abstract) [59]	76 healthy resistance-trained men (29.9 ± 9.3 years)	RAN DB PLA PAR	PLA vs. 1 × 10^9^ CFU/day live *W. cogulans* GBI-30 6086 vs. 1 × 10^9^ inactivated *W. coagulans* GBI-30 6086 cells/day (14 days)	30 min cycling intervals followed by leg press (6 × 10 reps) and hex bar deadlifts (5 × 10 reps) at 65% 1RM and 5 × 20 reps of drop jumps	- 0, 0.5, 1, 2, 5, 24, 48, and 72 h after supplementation - Performance (countermovement jumps, isometric mid-thigh pull, Wingate anaerobic capacity) - Muscle damage (creatine kinase and myoglobin) - Inflammatory markers	- No differences (when compared to PLA) in performance as measured by: - Isometric mid-thigh pull - Isokinetic dynamometry (isometric peak torque, isokinetic peak torque, total work, and average power) - Countermovement jump performance (peak force, rate of force development)
Holley, 2023 (Abstract) [60]	76 healthy resistance-trained men (29.9 ± 9.3 years)	RAN DB PLA PAR	PLA vs. 1 × 10^9^ CFU/day live *W. coagulans* GBI-30 6086 vs. 1 × 10^9^ inactivated *W. coagulans* GBI-30 6086 cells/day (14 days)	30 min cycling intervals followed by leg press (6 × 10 reps) and hex bar deadlifts (5 × 10 reps) at 65% 1RM and 5 × 20 reps of drop jumps	- 0, 0.5, 1, 2, 5, 24, 48, and 72 h after supplementation - Muscle damage (creatine kinase and myoglobin) - Inflammatory markers (TNFα, IL6, IL10, CRP, MCP1) - Immune (cell counts)	- No differences (when compared to PLA) for cell counts, CRP, TNFα, and MCP1 for either heat-killed or live cells - IL-6 was greater in PLA vs. heat-killed cells (30 min and 2 h) and live cells (5 and 72 h) - IL10 was greater than PLA vs. heat-killed cells (5 and 24 h) and live cells (0, 2, 24, and 72 h)
Kalman, 2018 [61]	16 healthy males (22.6 ± 3.4 years)	RAN DB PLA PAR	PLA vs. *W. coagulans* GBI-30 6086 (28 days)	In vitro bacterial challenge 60 min of treadmill running at 60–80% HRR	- Basal immune (IgA, CBC), T cells, NK cells, cytokines, and cortisol	- No differences when compared to PLA - WBC ↑ WBC 10 min after Ex in StaImune - T cell counts and proportions improved in postbiotic condition - ↑ recovery for cortisol - No change in cytokine counts
Cheng, 2023 [62]	30 healthy males (20–25 years)	RAN DB PLA PAR	PLA vs. 3 × 10^10^ heat-killed *Lactiplantibaccilus plantarum* TWK10 cells/day (6 weeks)	No exercise intervention 30 min run at 60% VO_2_Max Run to exhaustion at 85% VO_2_Max	- Endurance exercise performance - Muscle mass and body composition - Muscle stress and fatigue	- Endurance time was increased in heat-killed TWK10 vs. placebo - Grip strength on both hands was increased in heat-killed TWK10 vs. placebo - Muscle mass was greater in heat-killed TWK10 - Lactate and ammonia were reduced in heat-killed TWK10 vs. PLA during exercise and recovery
Lee, 2022 [63]	53 healthy adults (20–30 years)	RAN DB PLA PAR	PLA vs. 3 × 10^11^ CFU/day live *Lactiplantibaccilus plantarum* TWK10 cells vs. 3 × 10^11^ heat-killed *Lactiplantibaccilus plantarum* TWK10 cells/day (6 weeks)	No exercise intervention Standardized bout of exercise was completed before and after supplementation	- Blood was collected before and after supplementation and throughout and in response to exercise challenge - Exercise performance was evaluated using running time to exhaustion at 85% VO_2_Max - Body composition via bioelectrical impedance analysis	- Live and heat-killed cells increased exhaustion times after supplementation; changes observed were greater than PLA - No difference in exhaustion times between live and heat-killed - Heat-killed TWK10 lowered inflammatory response to challenging exercise - Heat-killed did not impact body composition changes - Heat-killed TWK10 significantly changed beta-diversity vs. control
Sawada, 2019 [39]	49 male collegiate long-distance relay runners (18–22 years)	RAN DB PLA PAR	Daily intake of 200 mL beverage containing either PLA or 1 × 10^10^ *Lactobacillus gasseri* CP2305 (12 weeks)	Daily intake while training for and competing in All-Japan university championships	- Physical and mental health (fatigue scales, state/trait anxiety, Pittsburgh sleep quality, general health questionnaire, anxiety and depression scale) - Blood counts, hormones, damage markers - Fecal microbiota	- When compared to PLA, *Lactobacillus gasseri* CP2305 facilitated recovery from fatigue and relieved anxiety and depressive mood - Prevented reduction in hemoglobin - Facilitated increase in growth hormone - Increased alpha and beta diversity in fecal microbiome
Sashihara, 2013 [64]	44 healthy male university football club members (<30 years)	RAN DB PLA PAR	PLA vs. 100 mg/day of 1 × 10^10^ heat-killed *Lactobacillus gasseri* LG2809 cells vs. 100 mg/day heat-killed *L. gasseri* LG2809 cells + 900 mg/day *α-lactalbumin* (4 weeks)	60 min of strenuous cycle ergometer exercise at 70% heart rate reserve Completed before and after supplementation	- Workload and calories expended - Profile of Mood States questionnaires (baseline and 4 wks) - Visual analog scale for fatigue (PRE and 10 min post-exercise before and after supplementation) - Complete blood counts, NK cell activity, levels of reactive oxygen metabolites, TGF-β1, and cortisol	- Ingestion of *Lactobacillus gasseri* LG2809 did not affect exercise performance when compared to PLA - When compared to PLA, *Lactobacillus gasseri* LG2809 elevated depressive mood states and prevented a reduction in NK cell activity to strenuous exercise - *Lactobacillus gasseri* LG2809 cells + *α-lactalbumin* alleviated minor resting fatigue and reduced serum reactive oxygen metabolites and TGF-β1 levels
Komano, 2023 [65]	37 university long-distance track and field athletes (>18 years)	RAN PLA DB PAR	PLA vs. heat-killed dry *Lactococcus lactis* Plasma 1 × 10^11^ cells/day (14 days)	Days 1 to 14: performed sport-specific training under the supervision of the track and field coach Day 15: single 2 h exercise bout using a cycle ergometer at 70–80% of heart rate maximum	- Plasmacytoid dendritic cell maturation markers - Blood parameters - Physiological indices - Fatigue-related assessments: visual analog scale - Assessed on days 1 and 15 before and after exercise - Daily symptoms related to physical condition	- CD86 as a maturation marker on dendritic cells was significantly higher and cumulative days of fatigue were significantly fewer in the heat-killed dry *Lactococcus lactis* plasma group vs. PLA on day 15. - During day 15 exercise, fatigue parameters were significantly lower in the heat-killed dry *Lactococcus lactis* plasma group.

RAN = randomized; DB = double-blind; PLA = placebo-controlled; PAR = parallel group supplement assignment; ↑ = increased; ↓ = decreased.

## Data Availability

No new data were created from this paper. This systematic review was not publicly registered nor was any formalized protocol prepared.
